# Office‐Based Blue Laser Versus Coblation Therapy for Inferior Turbinate Hypertrophy: A Pilot Study

**DOI:** 10.1002/oto2.70127

**Published:** 2025-05-14

**Authors:** Abdul‐Latif Hamdan, Zeina Maria Semaan, Lana Ghzayel, Yara Yammine, Jonathan Abou Chaar, Jad Hosri, Patrick Abou Raji Feghali, Anne Marie Daou, Elie Alam

**Affiliations:** ^1^ Department of Otolaryngology–Head and Neck Surgery American University of Beirut Medical Center Beirut Lebanon

**Keywords:** blue laser, coblation, inferior turbinate hypertrophy, nasal obstruction, office‐based

## Abstract

**Objective:**

The aim of this pilot study is to compare the effectiveness of office‐based blue laser therapy with coblation therapy in patients with inferior turbinate hypertrophy (ITH).

**Study Design:**

Retrospective chart review.

**Setting:**

Tertiary referral center.

**Methods:**

Patients presenting with nasal obstruction between November 2022 and November 2024, and underwent coblation or blue laser therapy for turbinate reduction were reviewed. Demographic data included age, gender, smoking, history of allergy, history of reflux disease, and history of prior nasal surgery. All patients had filled the nasal obstruction symptom evaluation (NOSE) questionnaire and the visual analog scale (VAS) before and on follow‐up after treatment. Patient's level of comfort during the procedure was also rated using a 10‐point Likert scale with a higher score indicating a greater level of comfort.

**Results:**

A total of 10 patients underwent office‐based blue laser therapy for turbinate reduction, and 10 patients underwent office‐based coblation of the inferior turbinates. In the subgroup of patients who underwent office‐based blue laser therapy, the mean NOSE score and VAS score decreased significantly (*P* = .005). In the subgroup of patients who underwent coblation, the mean NOSE score and VAS score decreased significantly (*P* = .005). When comparing the two subgroups, the difference in the drop of the NOSE score was not statistically significant (*P* = .198). Similarly, the difference in the drop of VAS score was not statistically significant (*P* = .280).

**Conclusion:**

The results of this investigation indicate that both coblation therapy and blue laser therapy are effective office‐based treatment modalities in patients with ITH with comparable results.

Nasal obstruction refers to the sensation of inadequate airflow through the nasal passages, often described as nasal stuffiness or nasal block.^1^ It affects up to one‐third of the population with a higher prevalence noted in women compared to men.^2^, ^3^ Nasal obstruction may be bilateral or unilateral, acute or chronic, continuous or transient in nature. It can result from various anatomical, neurological, and/or iatrogenic factors. Among the frequently reported anatomical factors are septal deviation and inferior turbinate hypertrophy (ITH).^3^, ^4^ Common causes of ITH include allergic rhinitis, vasomotor rhinitis, and chronic hypertrophic rhinitis.^3^ ITH may also develop as a compensatory mechanism in response to a septal deviation, referred to as compensatory hypertrophy. Other less common causes of nasal obstruction include sinonasal polyposis, septal perforation, and tumors of the nasal and nasopharyngeal cavities.^4^, ^5^, ^6^

Treatment of ITH has been widely discussed in the literature. Affected patients are initially treated conservatively with medications like antihistamines, corticosteroids, and decongestants, with a success rate ranging from 23.7% to 62%.^7^, ^8^ In patients who experience minimal relief, surgical reduction of the inferior turbinates is advocated.^9^ The most commonly performed surgeries are total turbinectomy, partial turbinectomy, and submucosal turbinectomy. The main limitations of these surgeries are the need for general anesthesia, the risk of bleeding, and the prolonged recovery period.^10^, ^11^ With the reform in otolaryngology practice and the shift from the operating room to the clinic, many turbinate reduction procedures are now performed in an office setting under local anesthesia. Office‐based procedures include radiofrequency ablation (RFA), electrocautery, microdebrider‐assisted turbinoplasty, and laser‐assisted turbinoplasty.^9^ Both cutting and photoangiolytic lasers have been used such as the carbon dioxide CO_2_ laser, pulsed dye laser (PDL), potassium titanyl phosphate (KTP) laser, and more recently, the blue laser.^10^ The blue laser is a 445‐nm photoangiolytic laser with a hybrid property that allows both cutting and coagulation when used in a contact and noncontact mode. In 2024, the authors of this manuscript reported their experience with the blue laser in the management of 14 patients with ITH. All participants had a significant improvement following office‐based blue laser therapy, and the mean NOSE score decreased from 13.07 preoperatively to 2.64 postoperatively. Similarly, the VAS score for nasal obstruction dropped from 7.43 to 2.0 (*P* = .002). The procedure was tolerated well by the patients with no complications noted.^12^

The aim of this pilot study is to compare the effectiveness of office‐based blue laser therapy with coblation therapy in patients with ITH. The authors chose to use the blue laser because of its superior photoangiolytic and hemostatic properties compared to the PDL and KTP laser.^11^ The blue laser machine is lightweight and robust, which makes it easy to transfer from the operating room to the clinic. Moreover, the blue laser glass fibers come in different sizes and can be easily inserted into the nasal suction for usage.^13^ Patient self‐reported outcome measures were used to assess the success of treatment. The hypothesis set forth by the authors is that both treatment modalities are effective with similar results.

## Methods

After obtaining approval from the Institutional Review Board (IRB ID: BIO 2022‐0280), the medical records of patients presenting to a tertiary referral center with nasal obstruction between November 2022 and November 2024 were reviewed. Patients with persistent nasal obstruction despite medical therapy (at least 3 months of intranasal steroid and oral antihistamines) and with evidence of ITH for which they underwent office‐based blue therapy or coblation under local anesthesia were included. These were consecutive patients who failed medical therapy and agreed to undergo office‐based therapy. None of the patients had a prior history of nasal surgery or had been enrolled in a previous investigation. Agreeing to undergo office‐based therapy for ITH was the inclusion criterion. Patients who refused to undergo office‐based treatment and decided to undergo surgery in the operating room were not included in this study. Patients who presented to the first author of this manuscript were offered blue laser therapy, which was his line of expertise, whereas those who presented to the last author were offered coblation, which was his line of expertise. The stratification of the participants into the laser versus coblation groups was based on the surgeon's experience and no other selection criteria.

**Figure 1:**
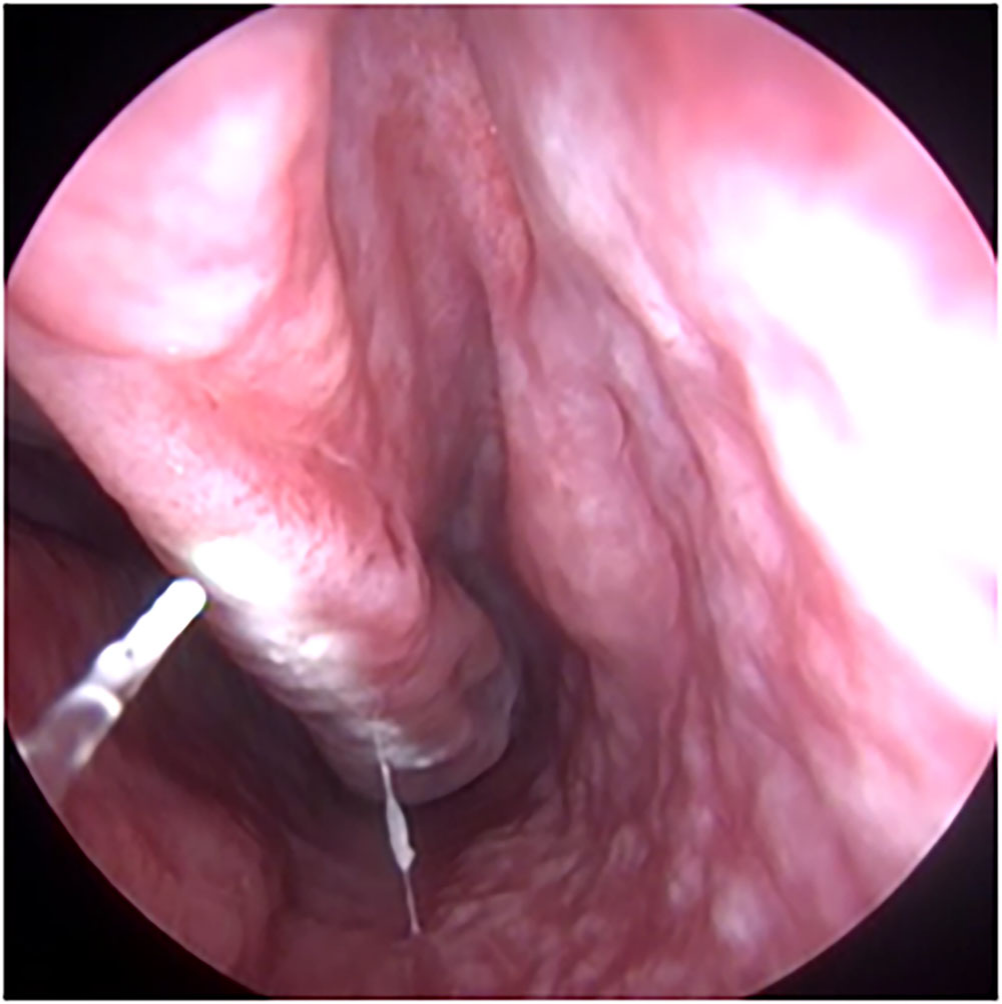
Intraoperative image showing horizontal line along the medial surface of the inferior turbinate simulating a zebra line following noncontact application of the blue laser.

Coblation of the inferior turbinate was performed using the REFLEX ULTRA™ 45 turbinate reduction wand at a power of 6 W. Lidocaine‐soaked pledgets and submucosal injections were used for anesthesia. Under endoscopic guidance, tissue ablation was achieved by activating the coblator for 10 seconds at multiple points along each inferior turbinate. The procedure included outfracturing the turbinate using a Cottle elevator. Laser inferior turbinate reduction was performed using the blue laser in contact and noncontact mode (A.R.C. Laser, Nuremberg, Germany; Figure 1). The laser was delivered using the 400‐µm glass fiber and using the following settings: power 10 W, pulse duration 10 milliseconds, and pulse pause 300 milliseconds. The presence of ITH was confirmed by endoscopic examination of the nasal cavity using a flexible nasopharyngoscope. Patients with a history of sinusitis and sinonasal symptoms other than nasal obstruction such as nasal discharge, postnasal drip, facial pain, and headache were excluded.

Demographic data retrieved from the patients' medical charts included age, gender, smoking, history of allergy, history of reflux disease, and history of prior nasal surgery. The two outcome measures used to gauge the improvement in nasal breathing were the nasal obstruction symptom evaluation (NOSE) scale and the visual analog scale (VAS) for nasal obstruction. All patients had filled the NOSE questionnaire and the VAS before and on follow‐up after treatment. The NOSE is a 5‐item questionnaire that rates the symptoms of nasal obstruction from 0, indicating no problem, to 4, indicating a severe problem with a maximum total score of 20.^14^ The VAS is a 10‐point scale for discomfort ranging from 0 to 10 with 0 indicating no discomfort and 10 indicating maximum discomfort. Patient's level of comfort during the procedure was also rated using a 10‐point Likert scale with a higher score indicating a greater level of comfort.

### Statistical Analysis

Categorical and continuous variables were described using frequencies and means (±standard deviation), respectively. Nonparametric data underwent analysis via the Wilcoxon signed‐rank test for intragroup comparison before and after intervention, and the Mann‐Whitney *U* test was used for intergroup comparison. Parametric data were analyzed using the paired samples *t* test for intragroup comparison and the independent sample *t* for intergroup comparison. The chi‐square test was used for the comparison of proportions between categorical variables. All analyses were conducted using the Statistical Package for the Social Sciences (SPSS) version 29 software package. A two‐tailed *P*‐value < .05 was considered statistically significant.

## Results

### Demographic Data

A total of 20 patients were included in this study. Ten patients underwent office‐based blue laser therapy for turbinate reduction, and 10 patients underwent office‐based coblation of the inferior turbinates. The male‐to‐female ratio was 3:2, and the mean age of the total study group was 34.45 ± 9.55 years. Of the 20 patients, 12 had a history of smoking, 8 had a history of allergy, and 4 had a history of reflux disease. There was no significant difference in the prevalence of smoking, history of allergy, and history of reflux disease between the two groups (*P* > .05). Seven patients had a history of nasal surgery. See Table 1.

**Table 1:** Demographic Data of the Study Population

Demographic data	Total study group (N = 20)	Blue laser (N = 10)	Coblation (N = 10)	*P*‐value
Gender (male:female)	3:2	3:2	3:2	1.00
Age, y (mean ± SD)	34.45 ± 9.55	33.10 ± 11.65	35.80 ± 7.25	.542
Smoking	12 (60)	5 (50)	7 (70)	.650
Allergy (n (%))	8 (40)	4 (40)	4 (40)	1.00
Reflux disease (n (%))	4 (20)	3 (30)	1 (10)	.582
History of nasal surgery (n (%))	7 (35)	3 (30)	4 (40)	1.00

### The Mean NOSE Scores Preoperatively and Postoperatively in Both Subgroups

All patients who underwent office‐based blue laser therapy for turbinate reduction tolerated the procedure well and had no complications. The mean NOSE score decreased significantly from 16.00 ± 2.26 preoperatively to 2.60 ± 2.71 postoperatively (*P* = .005). All patients had a drop in their NOSE scores following laser therapy.

In the subgroup of patients who underwent coblation of the inferior turbinates, the mean NOSE score decreased significantly from 17.70 ± 0.94 preoperatively to 5.50 ± 3.68 postoperatively (*P* = .005). All patients had a drop in their NOSE scores following coblation therapy. See Table 2.

**Table 2:** Mean Nasal Obstruction Symptom Evaluation Score Before and After Blue Laser and Coblation Therapy

	Before	After	*P*‐value
Blue laser (n = 10)	16.00 ± 2.26	2.60 ± 2.71	.005*
Coblation (n = 10)	17.70 ± 0.94	5.50 ± 3.68	.005*

* Statistically significant *p* < .05.

### The Mean VAS Scores Preoperatively and Postoperatively in Both Groups

In the subgroup of patients who underwent office‐based blue laser therapy for turbinate reduction, the mean VAS score for nasal obstruction decreased significantly from 8.00 ± 1.56 preoperatively to 1.70 ± 2.16 postoperatively (*P* = .005). All patients had a drop in their VAS scores following laser therapy.

In the subgroup of patients who underwent coblation of the inferior turbinate, the mean VAS score for nasal obstruction decreased significantly from 8.05 ± 1.08 preoperatively to 3.30 ± 2.05 postoperatively (*P* = .005). All patients had a drop in their VAS scores following coblation therapy. See Table 3.

**Table 3:** Mean Visual Analog Scale Score Before and After Blue Laser and Coblation Therapy

	Before	After	*P*‐value
Blue laser (n = 10)	8.00 ± 1.56	1.70 ± 2.16	.005*
Coblation (n = 10)	8.05 ± 1.08	3.30 ± 2.05	.005*

* Statistically significant *p* < .05.

### Difference in the Drop of the Mean NOSE and VAS Scores Between the Two Subgroups

**Figure 2:**
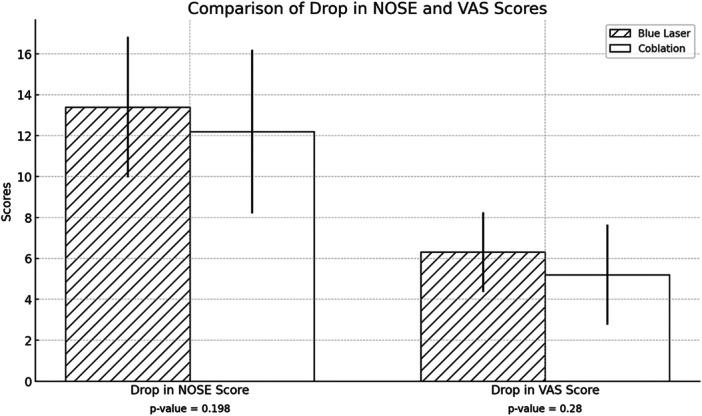
Histogram showing the difference and standard error in the drops of the nasal obstruction symptom evaluation (NOSE) and visual analog scale (VAS) scores between blue laser and coblation.

When comparing the two subgroups, blue laser therapy versus coblation therapy, the difference in the drop of the NOSE score was not statistically significant (*P* = .198). Similarly, the difference in the drop of VAS score was not statistically significant (*P* = .280; Figure 2).

### Level of Comfort During the Procedure

The procedures were well tolerated with a completion rate of 100%. The level of comfort ranged between 3 and 10 with a mean of 7.25 ± 2.24. The mean score of comfort in the laser subgroup was 7.60 ± 2.22. The mean score of comfort in the coblation group was 6.90 ± 2.33. The difference in the level of comfort between the two subgroups was not statistically significant (*P* = .436).

## Discussion

Several treatment modalities for inferior turbinate reduction have been described in the literature. In this study, the treatment modalities used were coblation and blue laser therapy. Coblation is a minimally invasive technique that uses low‐temperature radiofrequency energy to dissolve targeted soft tissue, making it effective for inferior turbinate reduction. Performing this procedure in an office setting offers several advantages such as minimal postoperative discomfort and a quicker recovery, making it a more convenient and cost‐effective option for patients. The results of this investigation showed that all patients had improvement in breathing with a decrease in the NOSE score and decrease in VAS score for nasal obstruction. These findings are in accord with the literature. In a study that included 26 patients with ITH treated with coblation, Bhattacharyya and Kepnes reported significant improvement in nasal obstruction with minimal complications. There was a significant decrease in the nasal obstruction and overall nasal symptoms domains of the Rhinosinusitis Symptom Inventory (RSI) at 3 months postprocedure (*P* = .018 and *P* = .015, respectively) and 6 months postprocedure (*P* < .001 and *P* < .001, respectively). With respect to the short nasal symptom questionnaire, the authors reported a significant decrease in nasal obstruction and amount of time with nasal obstruction at the 3‐month interval (*P* = .006 and *P* = .011, respectively) and at the 6‐month interval (*P* = .001 and *P* = .006).^15^ Lee et al evaluated the effectiveness of combining coblation‐assisted turbinoplasty with septoplasty in 21 patients with nasal septal deviation and severe ITH. Postoperative assessments showed significant improvement in nasal cavity volume (*P* < .05) and minimal cross‐sectional area as measured by acoustic rhinometry (*P* < .05). Additionally, patients reported substantial relief in nasal obstruction symptoms, as indicated by improved VAS scores over a 1‐year follow‐up period (*P* < .05).^16^ In a systematic review by Hytönen et al that included 1020 patients, the authors evaluated the efficacy and safety of RFA in treating nasal obstruction using the VAS and quality of life (QoL) questionnaire. Results showed that RFA significantly improved nasal airflow and reduced nasal obstruction symptoms (*P* < .05). The procedure was generally well tolerated, with only minor complications noted such as mild discomfort, edema, and crusting, which resolved within a month.^17^

Another alternative for the treatment of ITH in an office setting is laser inferior turbinate reduction. This procedure has also been proven to be effective in alleviating nasal obstruction with a success rate that varied between 70.8% and 85.4% depending on patient demographic characteristics and type of laser used.^18^, ^19^, ^20^, ^21^, ^22^ The main advantages of laser inferior turbinate reduction are reduced risk of bleeding, less pain, and a faster recovery period.^21^ The results of this investigation showed significant improvement in patient‐reported outcome measures following blue laser therapy, which is in accord with the literature. Supiyaphun et al reported their experience with KTP laser inferior turbinoplasty in 48 patients and noted improvement in nasal obstruction in all the participants with a cure rate ranging from 70.8% to 77.1%. The subjective improvement was associated with a significant increase in inspired nasal airflow and nasal cavity volume (*P* < .001).^18^ In a study on 306 patients with ITH treated using the CO_2_ laser, Testa et al reported significant improvement in nasal flow, symptoms, and QoL, with sustained long‐term efficacy (*P* < .01).^19^ In a prospective clinical trial on 42 patients with therapy‐refractory rhinitis medicamentosa, Caffier et al noted that diode laser inferior turbinate reduction led to significant improvement in nasal airflow (250.4‐413.9 cm³/s at 150 Pa). Moreover, 88% of the study population stopped decongestant abuse after 6 months (74% after 1 year).^20^ In a study on 62 patients with chronic nasal obstruction who underwent diode laser‐assisted turbinate reduction, Cakli et al reported a significant improvement in VAS scores and a significant increase in nasal patency (*P* < .001). The authors also noted an 85.4% patient improvement rate in nasal breathing after 1 year.^21^ Hussain and Ahmad conducted CO_2_ laser turbinoplasty on 53 patients and noted significantly improved NOSE scores from 64.90 to 5.09 and VAS scores from 6.05 to 1.18 at 3 months with minimal postoperative pain, good tissue healing, and reduced medical morbidity.^22^

The main finding in our study is the comparable results of coblation and blue laser therapy of ITH. The lack of significant difference in the outcome measures between the two subgroups suggests that both treatment alternatives are equally effective and can be used interchangeably in the clinic. These results are interesting knowing that the blue laser is a photoangiolytic laser that is absorbed by oxyhemoglobin and hence targets selectively the submucosal vasculature, whereas coblation therapy targets the submucosal vasculature and surrounding erectile tissues as well. Although the results of this investigation are novel and interesting, the reader should keep in mind the limited depth of penetration of the blue laser, which does not exceed 3 to 4 mm at high power (10 W) and remains a constraint in addressing the deep tissues of the inferior turbinate.^11^ Potential complications include inadvertent skin burns and injury to vital structures can occur, and precautionary measures such as using goggles are advocated.

This study suggests that blue laser therapy could be an alternative therapy for ITH. The blue laser is easy to handle, and the learning curve for turbinate reduction is not steep, thus allowing young otolaryngologists and residents to use it in an office setting. Nevertheless, this study has its limitations, namely the small sample size. A power analysis was conducted using effect sizes derived from previous studies on similar interventions. The lack of significant findings in this investigation could be attributed to the small sample size. Future studies with a larger sample size will help validate these findings and further clarify potential differences in clinical outcomes. And second is the relatively short follow‐up period. However, as a pilot study, it alludes to the significant role of blue laser in the management of patients with ITH in an office setting.

## Conclusion

This study is the second study in the literature on the use of blue laser for the treatment of ITH. The results of this investigation indicate that both coblation therapy and blue laser therapy are effective office‐based treatment modalities in patients with ITH with comparable patient‐reported outcome measures. These findings widen the scope of application of blue laser as a photoangiolytic laser in the management of nasal obstruction. Blue laser can be added to the armamentarium tools used in the office for the treatment of ITH. Nevertheless, future prospective studies with a larger sample size and longer follow‐up are needed to establish the duration of improvement posttreatment.

## Author Contributions

**Abdul‐Latif Hamdan**, study design and manuscript writing – original draft; **Zeina Maria Semaan**, data collection and manuscript writing – original draft; **Lana Ghzayel**, data collection and formal analysis; **Yara Yammine and Jonathan Abou Chaar**, literature review and manuscript writing – original draft; **Patrick Abou Raji Feghali and Anne Marie Daou**, manuscript writing – review and editing; **Elie Alam**, study design and critical review.

## Disclosures

### Competing interests

The authors declare that there is no conflict of interest.

### Funding source

None.
